# “I couldn’t”: A phenomenological exploration of ethical tensions experienced by bereaved family members during the pandemic

**DOI:** 10.1080/17482631.2023.2186337

**Published:** 2023-03-15

**Authors:** Melanie Vachon, Alexandra Guité-Verret, Deborah Ummel, Dominique Girard

**Affiliations:** a Psychology Department, Université du Québec à Montréal, Montréal, Canada; b Center for Research and Intervention on Suicide, Ethical Issues and End-of-Life Practices (CRISE), Montreal, Canada; c Réseau Québécois de Recherche en Soins Palliatifs et de fin de vie (RQSPAL), Montréal, Canada; d Psychoeducation Department, Université de Sherbrooke, Longueuil, Canada; e Pain and Palliative Medicine Department, Radboud University Medical Center, Nijmegen, The Netherlands

**Keywords:** Bereavement, ethics, dilemma, family caregivers, moral distress, phenomenology, pandemic

## Abstract

**Introduction:**

The COVID-19 pandemic entailed significant changes in accompaniment, end-of-life, and bereavement experiences. In some countries, public health measures prevented or restricted family caregivers from visiting their dying loved ones in residences, long-term care institutions, and hospitals. As a result, family members were faced with critical decisions that could easily lead to ethical dilemmas and moral distress.

**Aim:**

This study aimed to understand better the experience of ethical dilemmas among family caregivers who lost a loved one.

**Methds:**

We interviewed twenty bereaved family caregivers and analysed their narratives using Interpretative phenomenological analysis.

**Results:**

Our analysis suggests that family caregivers struggled with their multiple responsibilities (collective, relational, and personal) and had to deal with the emotional cost of their choices. Results display three emerging themes describing the experience of ethical struggles: (1) Flight or fight: Struggling with collective responsibility; (2) Being torn apart: Assuming relational responsibility and (3) “Choosing” oneself: The cost of personal responsibility.

**Discussion/Conclusion:**

Results are discussed and interpreted using an ethical, humanistic, and existential conceptual framework.

## Introduction

The COVID-19 pandemic entailed significant changes in accompaniment, end-of-life, and bereavement experiences. Numerous individuals who have contracted the virus have experienced suffering and isolation at the end of life. Also, end-of-life during the pandemic has been described as violent, sudden, and preventable (Breen et al., 2022; Kokou-Kpolou et al., 2020; Stroebe & Schut, 2021; Wang et al., 2020). Furthermore, in the early phases of the pandemic, family members were prohibited from visiting their dying loved ones because of strict public health measures (Vachon et al., 2020). In Quebec, Canada, on March 14th, 2020, the Prime Minister declared a state of public health emergency on the province territory and several measures to prevent virus transmission. Among those, family and voluntary caregivers were banned from visiting hospital centres and different types of residential aged care facilities until May 2020. This ban had a considerable deleterious effect on the accompaniment at the end-of-life and the bereavement trajectory of family caregivers (Vachon et al., 2020). Public health measures have also impacted funeral ceremonies and other commemorative rituals, for instance, the prohibition of any indoor or outdoor gathering. Funerals rituals were firstly strictly reserved for a “familial bubble.” Later, these measures have become more flexible, with several persisting constraints such as a limited number of people (e.g., only 25 people in a red zone), the requirement to wear a procedure mask, and to keep a distance of two metres at all times between individuals who were not living at the same address.

Previous research has emphasized that these tragic circumstances surrounding death have undermined families’ capacity to make meaning of their loss (Neimeyer et al., 2021). Most family caregivers experienced the inability to accompany their loved one at the end of life in the way they wanted and expected, and the inability to establish sufficient communication with their loved one and the healthcare team (Borghi & Menichetti, 2021; Cipolletta et al., 2022; Hanna et al., 2021; Pattison, 2020). As a result, most family caregivers did not witness nor understand the final trajectory of their loved one’s life (Guité-Verret et al., 2021). Metaphorically, family caregivers were deprived of the first chapters of the story of their grief: the preparation for death, the accompaniment, the acknowledgement of death by seeing the dead body and understanding its exact causes, and funeral rituals.

This overall inability to act and be present generated great suffering, including regrets, guilt, confusion, helplessness, death anxiety, anger, and a sense of injustice (Cipolletta et al., 2022; Dennis et al., 2022; Hernández-Fernández & Meneses-Falcón, 2022; Ummel & Vachon, 2022). Some studies suggest that these emotions may have impacts on the bereavement process, especially in terms of narrative activity and the search for meaning (Breen et al., 2022; Neimeyer & Lee, 2022). Therefore, on a narrative and experiential level, pandemic grief appears to be complex, “fragmented,” or “interrupted” (Guité-Verret et al., 2021; Mortazavi et al., 2021; Ummel & Vachon, 2022).

### Ethical dilemmas and moral distress

Given the critical care conditions under which the pandemic unfolded, most family caregivers could not act on what they knew to be right. Indeed, they faced critical decisions that could easily lead to ethical dilemmas and moral distress. In scientific literature, suffering from ethical dilemmas is often referred to as “moral distress” (Deschenes et al., 2020). Moral distress occurs when a person faces two equally important obligations and does not know what to do, as these obligations support mutually inconsistent (or mutually different?) courses of action (Deschenes et al., 2020; Jameton, 1984). Moral distress often occurs in uncertain situations when it is impossible to know the right course of action (Campbell et al., 2016). Facing ethical dilemmas can mean experiencing a violation of one’s integrity and core ethical values (Campbell et al., 2016), which seems even more true during the COVID-19 pandemic (Stroebe & Schut, 2021). This may be exacerbated for family caregivers, for whom the inability to act by one’s values and aspirations is associated, in normal times, with experiences of powerlessness, guilt, doubt, and vulnerability (Ullrich et al., 2020; Weigel, 2019). Furthermore, when it comes to grief, we know that the aftermath of the actions in response to ethical dilemmas can generate moral distress and alter both the integration and the meaning of the loved one’s death (Doka, 2005).

During the pandemic, most family caregivers experienced the brutal loss of the loved one and the inability to really *be there* for him or her. The impossibility of caring for their loved one at the end of life may have prevented them from following their ethics of care and may interfere with meaning-making during bereavement (Totman et al., 2015; Vachon, 2020b; Wilson et al., 2016). Indeed, prior studies’ results suggest that family caregivers’ experience and suffering during COVID-19 may be understood in light of ethical dilemmas. Many family caregivers struggle to balance two core values (i.e., protection and relation), while also trying to uphold the values of end-of-life care (e.g., compassion, relation, dignity, and meaning) (Perron et al., 2022). A conflict could be experienced between fear of and need for others (Mortazavi et al., 2021). Many family caregivers also perceived their experience as part of a collective sacrifice rooted in values of solidarity (Dennis et al., 2022; Parisi et al., 2021), which could also be a source of moral ambiguity. It seems more broadly that end-of-life accompaniment was more in line with public health restrictions than family caregivers’ needs and values (Ummel & Vachon, 2022).

This picture echoes the ethical struggles of healthcare professionals during the pandemic (Anderson-Shaw & Zar, 2020; De Brasi et al., 2021; Giannetta et al., 2021; Wiener et al., 2021). On the one hand, healthcare professionals wanted to act responsibly, be fair, respect the patients’ right to dignity and respect the families’ right to be with their dying relatives. On the other hand, they needed to control the risk of infectious transmission, work with scarce resources and respect the public’s interest in social distancing (Bustan et al., 2020). These results may provide insight into the essence of ethical dilemmas during COVID-19, which we summarize as the attempt and failure to combine virtue and utilitarianism (Perin & De Panfilis, 2021; Robert et al., 2020). However, whereas some authors have documented moral distress experienced by healthcare professionals during the pandemic, we found no research explicitly addressing the ethical and moral dilemmas that bereaved family caregivers may have experienced. Knowing that moral and ethical struggles may interfere with the bereavement process, such an in-depth understanding may provide clinical insights into the care of grieving patients.

### Aims

This study aims to provide an in-depth understanding of family caregivers’ experiences of ethical dilemmas during the COVID-19 pandemic. This study is part of a larger participatory research project aiming to better understand the experience of individuals who had lost a loved-one during the pandemic (Ummel et al., 2022; Vachon et al., 2020). The preliminary analysis allowed us to shed light on the numerous and profound ethical dilemmas experienced by participants. Therefore, we conducted supplementary analyses to deepen our comprehension of these dilemmas.

### The theoretical and methodological approach

This study was conducted through a phenomenological, existential, and hermeneutic approach (Smith et al., 2009) set within a constructivist (or interpretative) framework (Ponterotto, 2005). As constructivist researchers, we adhere to a relativist position that assumes multiple and equally valid realities (Ponterotto, 2005). We argue that meaning comes from the unique interactive dialogue between researchers and participants (Ponterotto, 2005).

Interpretative Phenomenological Analysis (IPA) provided us with a theoretical and methodological framework (Smith et al., 2009). IPA aims to describe, interpret, and situate the participants’ lived experiences (Smith et al., 2009; Tuffour, 2017). The interpretive focus of IPA is called “double hermeneutic”, referring to the active role of both the researcher and the participant during interpretation: the participant is trying to make sense of what is happening to him or her (during the interview), and the researcher is then trying to make sense of the participant’s sense-making (during the interview and data analysis; Smith, 2007). The researchers’ involvement requires an attitude of (self) reflection. In line with the hermeneutic tradition of phenomenology, this attitude is led by “a process of retaining wonder and openness to the world while reflexively restraining pre-understandings” (van Wijngaarden et al., 2017, 1741).

This research position shapes our understanding of ethical dilemmas as a human phenomenon. We argue, first, that ethical reasoning depends on personal relationships (e.g., relation, history, and conflict with the deceased, family, and friends), personal qualities (e.g., values, beliefs, insights, awareness, feelings, previous experiences), and social, historical, and cultural context (e.g., stressors, social and community support, social attitudes towards death, public health measures). Second, we argue that ethical experiences and decisions steam from a co-construction of meaning between patients, families, and clinicians but that the COVID-19 pandemic disrupted the relationship of care that typically generates this co-construction (Manara et al., 2014). Finally, we argue that the reasoning about ethical values and responsibility is not immediate, obvious, or definite; its meaning is constructed and changes according to the context and the dialogue in which it takes place.

### A relational and narrative approach to ethics in the contexts of family caregiving and bereavement

From this standpoint, we propose a relational and narrative approach to ethics in the context of family caregiving and bereavement. Ethics starts in the context of narratives and relationships (Ricoeur, 1995). The key question in such ethics is less “Can *I* live with this?” than “Can *we* live with this?” (Abma, 2005). Therefore, the answer to the latter question are co-created and socially contingent. Facing dilemmas, people are always “situated” in the world and have to make sense of both this world and their existential situation. However, as already mentioned, most family caregivers were isolated during the pandemic and could not embrace the relational process through which ethical dilemmas are typically addressed.

Therefore, as part of our participatory research project, we created a space for a co-construction of meaning between bereaved family caregivers and researchers so the story of the death (and the various dilemmas that concerned it) could be socially narrated, shared, and better understood. Such interaction with participants concerned grief and the search for meaning: reflecting together on ethical dilemmas was part of an attempt to co-construct a more “viable narrative frame” of the loss (Neimeyer et al., 2014).

## Methods

### Participants and recruitment

The study sample included 20 participants (see Table I) living in the state of Quebec, Canada. The bereaved were first invited to participate in the study via the project’s Facebook page and the author’s contact networks or later recruited, thanks to chain-referral (snowball) sampling. For the present study, eligibility criteria included (a) being no younger than 18 years old, (b) having accompanied and experienced the loss of a loved one during the COVID-19 pandemic, and (c) having been confronted with ethical dilemmas related to his or her end of life. All participants had lost a loved one during the first wave of the pandemic (spring 2020), except two participants for whom it was during the second wave (fall 2020) and the third wave (spring 2021). The first contact with the participants took place on average three months after the death of the loved one, between May 2020 and March 2021.

**Table I.** Participants’ characteristics.

	N	M
**Age** (range 73–21)		54
**Gender**		
Women	19	
Men	1	
**Time since death** (range 12–260 days)		89
**Relationship with the deceased**		
Mother	8	
Father	7	
Sibling	1	
Spouse	3	
Grandparent	1	
**Place of death**		
Hospital	14	
Residence of elderly	6	

### Data collection

Qualitative data were collected in the form of personal narratives. Each participant was interviewed in private by videoconference, and the interview’s length ranged from 45 to 90 minutes. The number of interviews varied between one and five (*M* = 2), reliant on the participant’s individual needs. All interviews were audiotaped, transcribed verbatim, and verified (Kvale, 2007).

Following IPA guidelines, questions were open-ended, and the content was co-constructed. Participants were first encouraged to develop their stories from the following question: “Can you tell me what happened to you since the moment you suspected the viral contamination of your loved one until today?” During interviews, phenomenological prompts were used to deepen the exploration and nourish the interaction (e.g., what was it like? how did/do you feel? how did/do you understand this? what was difficult about this?). We also encouraged participants to narrate their stories to establish coherence between key events (e.g., what happened then?) such as the COVID-19 diagnosis, the circumstances surrounding death, and the mourning rituals.

Data collection was done in coherence with a relational and narrative approach to ethics. Individual interviews could help participants to develop a relational narrative that includes an understanding of ethical challenges. Indeed, how people narrate their experiences reveals to whom they feel responsible (Abma, 2005).

### Ethical considerations

This study received the approval of ethics committee at the university where the research took place (Université du Québec à Montréal, nº 2020–2590). All participants were given a full explanation of the project’s purpose and the voluntary nature of their participation. They were also assured of confidentially and integrity. Written or audiotaped consent was obtained for each participant. For relational and ethical reasons, we also paid particular attention to how the bereaved participants shared their experiences of suffering (Bourgeois-Guérin & Beaudoin, 2016).

### Data analysis

IPA is a flexible and creative, flexible and iterative process (Smith et al., 2009). The “hermeneutic circle” is perhaps the most resonant idea of it. The process of interpretation happens through thinking, digging, and connecting (Smith, 2007). With this in mind, the IPA guideline (Smith et al., 2009) involves iterative steps: (1) start data immersion by reading the first case many times and taking detailed reflexive notes, (2) develop emergent themes (meaning units) and associate them to participant’ quotes, (3) search for connections across the emergent themes (meaning units organization), (4) repeat step 1 to 3 with each case, (5) look for patterns across cases and make sure that they represent all participants, and (6) present a general structure of meaning (rather than fragmented results such as separate “categories” (van Wijngaarden et al., 2017)).

If IPA is strongly idiographic, it is thus concerned with the dynamic relationship between the part and the whole and, at another level, between the transcript and the researchers’ interpretation (Smith, 2007). Identifying units of meaning in IPA is a gradual process involving multiple levels of interpretation. As Smith (2007) puts it, IPA researchers must see “beyond” the first emerging themes to give access to the latent meaning, which nevertheless appears and connects with the manifest meaning. It is also to be understood that IPA findings are continuously open to new insights and reinterpretation because the participants’ experiences are never thoroughly explored and described (Smith et al., 2009). The method starts from the presupposition that meaning is infinite, expandable, and expanding (Dahlberg, 2006 cf. Merleau-Ponty, 2013).

### Quality and rigor

Our findings must be appreciated as phenomenological insights about a constructivist paradigm. The quality of interpretative phenomenological research relies on reflexivity and authenticity, which involves acknowledging (instead of ignoring) our subjectivity (Morrow, 2005). The quality of interpretative phenomenological research also relies on the meaning that emerges from the participant’s narratives (van Wijngaarden et al., 2017), which involves cultivating our capacity for resonance (Medico & Santiago Delefosse, 2014). Therefore, we proceed with our investigation based on our experiential and theoretical sources of knowledge while being reflexively aware of our methodological, theoretical, and ontological assumptions (Tracy, 2010; Willig, 2012). We followed Tracy’s (2010) eight quality criteria in qualitative research, as shown in Table II.

**Table II.** Tracy’s eight criteria of quality in qualitative research.

Criteria for quality	Means and practices through which criteria were achieved
Worthy topic	Relevance with regard to societal events and priorities In line with recent literature on caregiving and bereavement
Rich rigor	In-dept interviews and high level of transcription details Abundant and complex data Appropriate and complex theorical constructs Transparency about data collection and data analysis Peer-discussions to deepen data analysis
Sincerity	Self-reflexivity about pre-conceptions and experiences (e.g., reflexive diary and peer-discussions) Transparency about methodological and theoretical assumptions Recognition of the study limitations
Credibility	Thick descriptions (e.g., experientially rich descriptions, in line with data complexity) Immersion in the data to ascertain tacit knowledge Data crystallization (i.e., multiple data sources and multiple researchers, thanks to the embedment of the study within a wider project)
Resonance	Evocative narratives leading to empathic resonance Transferable findings
Significant contribution	Practical and heuristic significance (e.g., extending phenomenological knowledge on ethical dilemmas in the contexts of family caregiving and COVID-19 pandemic) Can lead to improve clinical practices
Ethics	Procedural ethics Situational ethics (e.g., reflexive and contextualized ethical practices) Relational ethics (e.g., availability of researchers throughout the participatory research project, and consideration of interdependence and co-construction between researchers and participants)
Meaningful coherence	Questions, paradigm, method and analysis in line with IPA Interconnections between stated goals, literature, data and interpretations

## Findings

Our analysis of the participants’ narratives allowed us to shed light on one essential theme: the experience of ethical tension. More precisely, this ethical tension emerged from the struggle to choose between two competing moral obligations. In all cases, forced choices came with emotional burden expressed by guilt, remorse, doubts, etc.

Ethical dilemmas and the resulting suffering were thus understood through three different experiential tensions of moral obligations: (1) Flight or fight: Struggling with collective responsibility; (2) Being torn apart: Assuming relational responsibility; (3) Choosing oneself: The cost of personal responsibility. If our analysis has led us to separate these themes, it is worth mentioning that participants dealt with ethical dilemmas in a complex, non-linear way. They often hesitated between two actions and changed their attitude after introspection or quick discussion with others. Our findings reflect how the participants focused on the unclear and uncertain aspects of their journeys, which were related to the dynamic relationship between their different responsibilities, and the decision-making context characterized by the urgency of the pandemic situation. The following section describes the three dynamic themes and elaborates on their complex articulation.

### Flight or fight: struggling with collective responsibility

During the first waves of the COVID-19 pandemic, confinement measures were stringent. For instance, family caregivers were prohibited to visit loved ones in elderly residences or health institutions. This rule was strictly implemented for end-of-life patients and patients actively dying from COVID-19. This policy was a turning point in the participants’ narratives, as it initiated an inner conflict between collective health demands and individual ethics. For most participants, health measures were linked to a common good but were also disconnected from an individual concern for others. A vast majority of participants recalled situations in which they felt powerlessness, anger, and deep anguish from the moment they could no longer visit (e.g., see, speak, touch, or connect with) their sick loved one. As they told us, they have been confronted with their loved one’s vulnerability or imminent death due to COVID-19. However, the participants felt powerless to preserve the comfort and dignity of their loved one or in offering security through compassionate care. This incapacity echoed the impossibility of acting responsibly towards the other, completely and authentically. In this context, participants had to reflect on their collective responsibility. They took a position on the restrictive policies: either chose to respect them with a significant amount of anxiety (flight) or fight them and find a way to visit their loved ones, which results in having no regrets.

For most participants, respecting collective health demands seemed to be part of collective responsibility. It was necessary but painful. Many revealed deep concerns about the hospitalization of their ill loved one, as it led to social interaction restrictions, and thus suffering and isolation:

The medical team told me on the phone to take my mother to the hospital as soon as possible. There’s guilt that goes with it … Because right after, I realized that I couldn’t get into the ambulance with her, or be in the hospital with her. I was not allowed to follow the ambulance, because on June 22, it was forbidden.

(P17)

For other participants who were allowed to visit their sick loved one in intensive care units, social interaction was also limited to meet collective health demands. Then, alongside the dying, there was an obligation to hold back affection and love as well as an obligation to rush farewell. One participant expressed how this conflict left everyone broken inside:

My grandmother was lying on her death bed and I couldn’t hug her. I put my arms around her, well, on her arms, because I wore a medical gown and gloves. Then she tried to hug me and I looked at her and I said “I can’t” … You could see that it was destroying her. That was also destroying me. Normally I would have hugged her, but at that time I would have caught COVID-19.

(P16)

After the loved one’s death, the practice of social distancing and compliance with confinement measures was not easy either. Some participants experienced conflict between collective responsibility and social expression of grief:

It is really hard for me to grieve. I couldn’t hug my children. I couldn’t cry in their arms … COVID-19 takes away all our values. It takes away everything. There is nothing we can do.

(P15)

For most participants, it largely seemed that social prohibitions felt like a painful cumulative effect of “I can’t”. In terms of lived experience, this addition meant an embodied impediment and, therefore, a reduction of the horizon of human possibilities.

Many participants “put their faith” in the medical system or public health policies as they felt powerless. They hoped hospitals and residential age care facilities would keep their loved ones safe and alive. After their loved one’s death, they seemed to “cling to the belief” that they acted correctly or did not choose wrong, even if they experienced ambivalence:

I spent days thinking: “What if I had tried to change the course of events?” You know people at the hospital, maybe you could have entered and seen mom? Maybe if you had seen her you could have influenced her care? Maybe the decline of her condition had something to do with her solitude.” That’s what’s on my mind right now. I don’t think it’s guilt. But you know, maybe I could have tried. Maybe I should have.

(P2)

Other participants did not believe in the medical system and instead fought against public health policies. Participants seemed to have rapidly established a hierarchy of values and responsibilities in those cases, placing collective responsibility after relational responsibility. Their “fight” seemed a way to regain some control (act more to suffer less) and to find meaning (e.g., to restore the loved one’s dignity or to go against a feeling of helplessness in the face of suffering):

I think there is no such thing as zero risk. I agreed with the rules, but still, I couldn’t. It was a matter of survival. I couldn’t leave my husband like this.

(P18)

On Sunday, I spoke to a nurse who dissuaded me from going there. She said: “You know the risks of being there?” I said: “Yes!” But then I panicked and thought: “Ok. I won’t go.” The day after that, I thought that it made no sense at all. I spoke to another nurse who told me to go see my mother. So I went on Monday. I went to see her. I will never regret that.

(P3)

My father was sitting on a chair, curled up. He was unable to talk. He was almost unconscious. It took him time to recognize me, because I wore a mask and everything. When the nurse went out of his room, I thought “Damn it!” and I took off everything. My father had to recognize me! I stroke his hair. I had took off my gloves. I wasn’t allowed to, but I didn’t care.

(P9)

At the last moment, some participants decided to act against their fears or against public health recommendations to see their dying loved one. Time was running out, and hesitancy was thus determinant. If many were grateful that their hesitancy finally resulted in meaningful acts (e.g., saying goodbye to the loved one, touching his or her hand, meeting his or her gaze, stroking his or her hair), others deeply regretted that their hesitancy prevented a last human connection:

I regret that I didn’t go see her on Friday. Because when I arrived at her bedside on Saturday morning, she couldn’t talk anymore. I mean, her eyes were shut and everything … She didn’t talk to me.

(P12)

I said to my mother: “I can’t take it any longer. I’m going to see him!” And I took the car right away. But fifteen minutes later, someone from the residence called us to say that my father had died … alone.

(P6)

The description and interpretation of this first theme led us to conceptualize the response to collective responsibility as a “fight or flight” response. This conceptualization allowed for a better understanding of how participants might have experienced their collective responsibility: their perception of the situation could lead to a flight-oriented attitude associated with feelings of helplessness and anxiety or a fight-oriented attitude, experienced as a source of opportunity, energy, and risk-taking.

### Being torn apart: assuming relational responsibility

Many ethical dilemmas were related to the participants’ whole relational landscape, including spouses, children, and other vulnerable persons who also needed care. In other words, participants’ experiences of responsibility were part of a complex interpersonal story. Indeed, for many participants, choosing not to visit their sick loved one and staying out of risk areas (e.g., hot zones in hospitals or residential age care facilities) was an attempt to stay healthy and not quarantine at home. That was important so they could continue to care for those around them who were still healthy, to whom they also felt responsible. Relational responsibility thus implies the difficulty of caring about and feeling responsible for their sick loved one while remaining present for other loved ones. If many participants finally decided not to visit their sick relative, this one was nevertheless in their minds and hearts:

It was frustrating, but at the same time, I understood. You know, the nurse was telling me that my father didn’t want us to put ourselves in danger. My father would have refused our help, knowing that I have two little girls and a husband.

(P1)

I didn’t want to go to a hot zone with inadequate mask and gloves. It made no sense. I was afraid of passing on the illness to my father’s wife, to people at work, to my neighbors … (P4)

The danger was real, and death was palpable for all participants and their family members. They learned through various media outlets that the pandemic was out of control, that outbreaks were frequently occurring, and that deaths were accumulating in hospitals and residential aged-care facilities. And so many participants rapidly felt a relational responsibility from the moment their relatives started to worry about them going to hot zones, revealing their mortality:

I have chronic asthma. And I am overweight … It was so hard you know because I had a choice to make. To go see my mother at the end of life or … My son looked at me, my little boy, who just turned ten years old, he looked at me, about to cry, and said: “I need you. I don’t want you to go there and die.”

(P8)

In other cases, participants were forced to choose between two relatives infected with COVID-19 or between two relatives living in different residential aged-care facilities:

I went to see my grandmother. She was feeling good, she had no symptoms. But at the same time, I couldn’t go see my dad, because if I went, you know the rules, I would have had to quarantine. I couldn’t see my dad.

(P9)

The experience of relational responsibility also involved a concern for the survivors. Some participants mentioned having to move from the present to the future to fulfill their responsibility for the living and the healthy. Future needs (e.g., funeral, family bereavement, family caregiving) were anticipated and considered an important part of the responsibility. For example, one participant faced a dilemma that led her to choose between visiting her unconscious dying dad at the hospital and staying with her healthy but vulnerable stepmother at home:

A volunteer from the residence told me: “If you go in, you’ll have to quarantine, and you’ll have to accept that, if your father dies, you won’t be there for your mother. So you have to choose: be on the side of the living, or on the side of the dead.” I chose not to go and he died two days later. What if I had gone … I sure think about it, but I shouldn’t. (P13)

The COVID-19 pandemic raised relational, existential questions that led to concrete ethical decisions: having to choose between the living and the dying. In this context, the responsibility of caring for the dying loved ones seemed to be irreconcilable with the responsibility of being present for the healthy loved ones, leaving some participants conflicted, frustrated and concerned.

### “Choosing” oneself: the cost of personal responsibility

Social restrictions imposed at the very beginning of the pandemic called for an ethical concern towards collective others. However, for most participants, it also called for an ethical recognition of the vulnerability of oneself and the community.

As they recounted their journeys, some participants mentioned their health problems and recalled feeling very concerned about those while they were struggling with their loved one’s health problems:

I wasn’t going to go visit him, and putting my health at risk. My health is fragile, you know…

(P6)

I have health problems myself. I have cardiac problems, and breathing problems. But I thought to myself: “Dad, it is not up to you to sacrifice yourself!”

(P1)

Facing multiple responsibilities (relational and personal) may mean sacrificing one person to save another (here, to save oneself, to survive). Some participants seemed to regret that self-responsibility could undermine acts of responsibility for others. At different levels, many participants acted in a self-protection manner or acknowledged their limits, while feeling moral distress.

You know, how far can you endanger yourself? How far can you … It’s this kind of conflict that I feel. It is far from simple … It is far from simple … (P20)

The noose is tightening, and tightening, and you feel a distress, a moral distress. What to do? (P19)

Self-protection often took on the function of protecting oneself against anxiety. For some participants, the idea of seeing a dying relative in a crisis was too confronting. They wanted to have a meaningful last moment with their loved one, consistent with their needs, but they felt that the context of his or her death would take them away from that “ideal”:

In this situation, you don’t even know if he will recognize us. We don’t know in what conditions he is. He won’t be able to talk. He is almost gone.

(P5)

I didn’t have the strength to be alone in there and see my father in this condition. (P13)

You know, the person who is sick, who suffers from Alzheimer, and who is on morphine, will not necessarily tell you like “I love you girl, I am proud of you, you are a good person”. Now, I deal with it. I can’t go back (P6).

In sum, it appeared that some participants felt vulnerable in the pandemic context and were not only concerned about the health of their sick loved ones but also their own. The desire to protect oneself (personal responsibility) seemed sometimes incompatible with the desire to be present for others (relational responsibility), generating moral distress.

To conclude, the results of this paper show the complexity of participants’ experiences. We have addressed the challenge of articulating one’s responsibilities in the face of ethical dilemmas in the COVID-19 pandemic. From a narrative point of view, this challenge was reflected in the participants’ difficulty in fluently expressing their responsibilities during the interviews and understanding their responsibilities during the pandemic crisis. Indeed, being confronted with their choices brought up nagging questions and intense suffering, including feelings of powerlessness and ambiguity. During their caregiving journey, participants seemed to believe they acted contrary to their values (e.g., to seek justice, to be present, to ease suffering, to respect the other’s existence, to cultivate self-respect).

## Discussion

According to existential phenomenology (Heidegger, 1962; Merleau-Ponty, 2013), human responsibility cannot be understood in another way than in the context of the whole existence and of our existence with others. Acting and understanding are about “being-in-the-world,” being with oneself and others in a specific cultural and historical frame.

Our phenomenological and interpretative analysis suggests that family caregivers recognized themselves and others when faced with ethical dilemmas. These findings highlight how collective, relational, and personal responsibilities implicate each other. During the COVID-19 pandemic, the participants seemed to be particularly aware of being in the world, so they tried to assume their multiple responsibilities, even if they were conflicting. This task was essential but impossible to accomplish, which has caused intense suffering among bereaved family caregivers. They have often sought a balance between macro and micro ethics and ultimately privileged one over the other. It appears that external and/or internal constraints led them to fail to act at the height of their moral convictions, if not to fail to perform the “right action completely.” This would have included acting by the relation between oneself, others, and the world, for example, to have the time to discuss the ethical issues with other people (including the loved one) to co-construct an ethical reflection and base the responsible act on a relationship.

The phenomenological work of Paul Ricoeur (2013) provides a better understanding of how participants suffered from these multiple and conflicting responsibilities. Ricoeur (2013) understands suffering as intersubjective powerlessness (for a conceptualization of suffering in the context of serious illness, see Daneault et al., 2022). More precisely, suffering is an incapacity to say, do, tell, and consider oneself a moral agent. Therefore, it comes with the awareness of self-limitation *vis à vis* oneself and others. Furthermore, according to Ricoeur, the suffering we experience from the suffering of others can be soothed by the attention we give them when we respond to their call for help (Furstenberg, 2021). With that in mind, responsibility is an ethical response to suffering and a way of surviving the powerlessness that comes with human suffering (Furstenberg, 2021). Responsibility is also part of an ethics of care based on interdependence and reciprocity since it emerges from belonging to a vulnerable, mortal humanity (De Panfilis et al., 2019; Vachon, 2020a).

Our analysis suggests that during the pandemic, family caregivers have been deprived of the possibility to act responsibly towards themselves and their loved ones, amplifying their suffering in the face of suffering and death. On the one hand, they were not able to be present enough to respond satisfactorily to the suffering of their loved one, nor were they able to return the love and attention that their loved one had given them in the past. On the other hand, the dying persons could not fulfill their final responsibility regarding transmission, legacy, and dialogue (Furstenberg, 2021; Vachon, 2014). In that respect, the context of the pandemic raised ethical dilemmas that, in turn, deprived families of the reciprocity that underlies ethical responsibility. Such reciprocity, as part of a meaningful connection with the other as death approaches (Holm et al., 2019; Otani et al., 2017; Pattison, 2020), is, however, important to provide a “good enough” accompaniment and create a sense of accomplishment that gives meaning to the life we had and the life we face now without the deceased (Bandini, 2020; Totman et al., 2015; Vachon, 2014, 2020b).

In line with existential and phenomenological theories, our study also supports the idea that overwhelming experiences, such as the death of a loved one, awaken existential consciousness (Yalom, 2008). As shown by our results, ethical dilemmas raised existential questions about life and death and existential distress, which led to a search for meaning and coherence. To the extent that ethical dilemmas took place in the context of death salience (Paul & Vasudevan, 2021) and often required a transition to end-of-life care, existential consciousness was heightened. In short, all participants were faced with (1) the suffering of the other, in front of which it was necessary but not always possible to do more, (2) their suffering in the face of human finitude, and (3) death. During the pandemic, they encounter their finitude: vulnerability to illness, suffering, and death. They also found themselves vulnerable to human responsibility as they experienced the difficulty of acting as an “agent of choice” (Bustan et al., 2020) and as a fundamental free being (Yalom, 1980), despite all contingencies.

An important contribution of this study is to underline that, in the context of the pandemic, responsibility towards oneself undermines responsibility towards others and that responsibility towards society (often understood as “the common good”) undermines existential and ethical responsibility. From this appeared an ethical crisis and a crisis of meaning in the face of suffering and death. If ethical responsibility usually is a constant movement between the self and the other (thinking of the self as another) (Ricoeur, 1995), this movement was interrupted during the pandemic. In light of these findings, we propose that the accompaniment at the end of life during COVID-19 was characterized by a reduction in its ethical scope and significance, consistent with reducing the possibilities to act as a human being. It seems to us that this reduction has fully participated in the impossibility of offering a “good death” to the loved one, then the difficulty of starting to grieve. Accompaniment was done at the rate of public health directives rather than in coherence with an ethics of care rooted in an existential responsibility. This upheaval in practices and values has led to intense suffering in the dying as much as intense moral suffering in those surviving. Our findings thus raise important questions, without providing definitive answers: how to adopt an ethical posture when the preservation of the humanity of the self does not correspond to the preservation of the humanity of others? How to adopt an ethical posture if the common good seems to be preserved at the expense of an ethic of care? How to care *for* and suffer *with* each other during a pandemic? How to choose between the dying and the living?

Finally, another significant contribution of this study is to underline how ethical dilemmas can affect the “work of grief” (Neimeyer et al., 2014). Ethical dilemmas (and the heartbreaking and troubling choices that arise from them) interfered with the active process of meaning reconstruction in the wake of loss and thus altered the integration of death. The presence of ethical dilemmas in the participants’ caregiving trajectory heightened their need to address the story of loss and place the events within the story of the relationship with the deceased, particularly the caregiving relationship. We perceived an increased need for coherence and co-construction of events. Indeed, even months after the death, most participants struggled to construct a viable narrative frame of the loss and the relationship due to ethical dilemmas. In this regard, the participatory nature of our research and the in-depth interviews we conducted seem to have allowed for a co-construction of meaning applicable to the work of grief. Moreover, ethical dilemmas often challenged participants’ life narratives and self-esteem, especially those who felt they had let their loved one down at the end of life. In this regard, our research set up a safe space open to moral suffering, focused on normalizing the difficulty of caring and acting ethically during the pandemic crisis.

### Methodological considerations and limitations

The evocative, dense, and complex narratives on which our analysis was based also extend the credibility of this study (Tracy, 2010) and may lead to empathic resonance (Tracy, 2010) and existential generalization (van Wijngaarden et al., 2017). As part of a participatory research project, this study also involved sharing the results with many participants, who were emotionally touched, and even grateful for the space this project created for the sharing and public recognition of their lived experiences. This resonance provided additional credibility to the study and demonstrated an ongoing relational ethic (Tracy, 2010).

Despite the contributions of this study, there were some limitations. First, participants’ narratives focused on events in 2020–2021, a time of exceptionally high public health measures and restrictions, such as visits to hospitals or elderly residences and funeral gatherings. Second, the sample was primarily female. However, it was representative given that most family caregivers are women (Washington et al., 2015). This study’s understanding must be appreciated about its historical, social, and interpretative context.

Finally, this study does not address the issue of the responsibility of families for the funerals (including honouring last wishes and caring for the dead body). Future studies may directly explore “funeral ethics” and how it may shape the experience of bereavement during the pandemic (Fery, 2020). Funeral ethics could be a final responsibility towards the other and the human community as it typically frames the reconciliation between death and the living (Bacqué, 2020).

## Conclusion

To our knowledge, this is the first study to focus directly on the ethical dilemmas experienced by family caregivers during the COVID-19 pandemic. The results offer an existential and phenomenological perspective, enriched by Ricoeur’s work on suffering and ethics. Our study may fill a gap in the current literature on the experience of bereaved family caregiving and allows for a direct expansion of the existing literature on ethical dilemmas related to the COVID-19 pandemic. The findings also capture the suffering that individuals may experience in the face of human responsibility to the living and the dead, to self and others, offering a non-pathological approach to grief and moral distress.

## References

[cit0001] Abma, T. A. (2005). Struggling with the fragility of life: A relational-narrative approach to ethics in palliative nursing. *Nursing Ethics*, 12(4), 337–12. 10.1191/0969733005ne799oa 16045242

[cit0002] Anderson-Shaw, L. K., & Zar, F. A. (2020). COVID-19, moral conflict, distress, and dying alone. *Journal of Bioethical Inquiry*, 17(4), 777–782. 10.1007/s11673-020-10040-9 33169271 PMC7652046

[cit0003] Bacqué, M. F. (2020). Place des funérailles dans le processus de deuil. *Jusqu’à la Mort Accompagner la Vie*, 140(1), 45–55. 10.3917/jalmalv.140.0045

[cit0004] Bandini, J. I. (2020). Beyond the hour of death: Family experiences of grief and bereavement following an end-of-life hospitalization in the intensive care unit. *Health*, 26(3), 267–283. 10.1177/1363459320946474 32748652

[cit0005] Borghi, L., & Menichetti, J. (2021). Strategies to cope with the COVID-related deaths among family members. *Frontiers in Psychiatry*, 12(622850), 1–4. 10.3389/fpsyt.2021.622850 PMC7946858 33716823

[cit0006] Bourgeois-Guérin, V., & Beaudoin, S. (2016). La Place de l’éthique dans l’interprétation de la souffrance en recherche qualitative. *Recherches Qualitatives*, 35(2), 23–44. 10.7202/1084379ar

[cit0007] Breen, L. J., Mancini, V. O., Lee, S. A., Pappalardo, E. A., & Neimeyer, R. A. (2022). Risk factors for dysfunctional grief and functional impairment for all causes of death during the COVID-19 pandemic: The mediating role of meaning. *Death Studies*, 46(1), 43–52. 10.1080/07481187.2021.1974666 34514956

[cit0008] Bustan, S., Nacoti, M., Borbol-Baum, M., Fischkoff, K., Charon, R., Madé, L., Simon, J. R., & Kritzinger, M. (2020). COVID-19: Ethical dilemmas in human lives. *Journal of Evaluation in Clinical Practice*, 27(3), 716–732. 10.1111/jep.13453 32929806

[cit0009] Campbell, S., Ulrich, C., & Grady, C. (2016). A broader understanding of moral distress. *The American Journal of Bioethics*, 16(12), 2–9. 10.1080/15265161.2016.1239782 27901442

[cit0010] Cipolletta, S., Entilli, L., & Filisetti, S. (2022). Uncertainty, shock and anger: Recent loss experiences of first-wave COVID-19 pandemic in Italy. *Journal of Community & Applied Social Psychology*, 0(0), 1–15. 10.1002/casp.2604 PMC9083240 35571876

[cit0011] Dahlberg, K. (2006). The essence of essences - the search for meaning structures in phenomenological analysis of lifeworld phenomena. *International Journal of Qualitative Studies on Health and Well-Being*, 1(1), 11–19. 10.1080/17482620500478405

[cit0012] Daneault, S., Azri, M., Ummel, D., Vinit, F., Côté, A., Leclerc-Loiselle, J., Laperle, P., & Gendron, S. (2022). Non-somatic suffering in palliative care: A qualitative study on patients’ perspectives. *Journal of Palliative Care*, 37(4), 1–8. 10.1177/08258597221083421 PMC9465553 35234108

[cit0013] De Brasi, E. L., Giannetta, N., Ercolani, S., Gandini, E. L. M., Moranda, D., Villa, G., & Manara, D. F. (2021). Nurses’ moral distress in end-of-life care: A qualitative study. *Nursing Ethics*, 28(5), 614–627. 10.1177/0969733020964859 33267730

[cit0014] De Panfilis, L., DiLeo, S., Peruselli, C., Ghirotto, L., & Tanzi, S. (2019). “I go into crisis when … ”: Ethics of care and moral dilemmas in palliative care. *BMC Palliative Care*, 18(70), 1–8. 10.1186/s12904-019-0453-2 31399094 PMC6689155

[cit0015] Dennis, B., Vanstone, M., Swinton, M., Brandt Vegas, D., Dionne, J. C., Cheung, A., Clarke, F. J., Hoad, N., Boyle, A., Huynh, J., Toledo, F., Soth, M., Neville, T. H., Fiest, K., & Cook, D. J. (2022). Sacrifice and solidarity: A qualitative study of family experiences of death and bereavement in critical care settings during the pandemic. *BMJ Open*, 12(1), 1–7. 10.1136/bmjopen-2021-058768 PMC8771806 35046010

[cit0016] Deschenes, S., Gagnon, M., Park, T., & Kunyk, D. (2020). Moral distress: A concept clarification. *Nursing Ethics*, 27(4), 1127–1146. 10.1177/0969733020909523 32249662

[cit0017] Doka, K. J. (2005). Ethics, end-of-life decisions and grief. *Mortality*, 10(1), 83–90. 10.1080/13576270500031105

[cit0018] Fery, B. (2020). Pour une éthique des funérailles en situation de pandémie. *Éthique Et Santé*, 17(3), 168–170. 10.1016/j.etiqe.2020.06.002 32837546 PMC7413651

[cit0019] Furstenberg, C. (2021). La Responsabilité et le rapport au temps selon Paul Ricoeur face à la mort: Résonnances en soins palliatifs. *Revue Internationale de Soins Palliatifs*, 3(35), 113–122. 10.3917/inka.213.0113

[cit0020] Giannetta, N., Villa, G., Pennestrì, F., Sala, R., Mordacci, R., & Manara, D. F. (2021). Ethical problems and moral distress in primary care: A scoping review. *International Journal of Environmental Research and Public Health*, 18(14), 1–14. 10.3390/ijerph18147565 PMC8303154 34300016

[cit0021] Guité-Verret, A., Vachon, M., Ummel, D., Lessard, E., & Francoeur-Carron, C. (2021). Expressing grief through metaphors: Family caregivers’ experience of care and grief during the Covid-19 pandemic. *International Journal of Qualitative Studies on Health and Well-Being*, 16(1), 1–13. 10.1080/17482631.2021.1996872 PMC8567898 34714218

[cit0022] Hanna, J. R., Rapa, E., Dalton, L. J., Hughes, R., McGlinchey, T., Bennett, K. M., Donnellan, W. J., Mason, S. R., & Mayland, C. R. (2021). A qualitative study of bereaved relatives’ end of life experiences during the COVID-19 pandemic. *Palliative Medicine*, 35(5), 843–851. 10.1177/02692163211004210 33784908 PMC8114449

[cit0023] Heidegger, M. (1962). *Being and time*. Blackwell Publishers.

[cit0024] Hernández-Fernández, C., & Meneses-Falcón, C. (2022). I can’t believe they are dead. Death and mourning in the absence of goodbyes during the COVID-19 pandemic. *Health & Social Care in the Community*, 30(4), 1220–1232. 10.1111/hsc.13530 PMC8444868 34363273

[cit0025] Holm, A. L., Severinsson, E., & Berland, A. K. (2019). The meaning of bereavement following spousal loss: A Qualitative study of the experiences of older adults. *SAGE Open*, 9(4), 1–11. 10.1177/215824401989427334290901

[cit0026] Jameton, A. (1984). *Nursing practice: The ethical issues*. Prentice Hall.

[cit0027] Kokou-Kpolou, C. K., Fernandez-Alcantara, M., & Cénat, J. M. (2020). Prolonged grief related to COVID-19 deaths: Do we have to fear a steep rise in traumatic and disenfranchised griefs? *Psychological Trauma: Theory, Research, Practice, and Policy*, 12(1), 94–95. 10.1037/tra0000798 32525367

[cit0028] Kvale, S. (2007). *Doing interviews*. Sage.

[cit0029] Manara, D. F., Villa, G., & Moranda, D. (2014). In search of salience: Phenomenological analysis of moral distress. *Nursing Philosophy*, 15(3), 171–182. 10.1111/nup.12048 24528533

[cit0030] Medico, D., & Santiago Delefosse, M. (2014). From reflexivity to resonances: Accounting for interpretation phenomena in qualitative research. *Qualitative Research in Psychology*, 11(4), 350–364. 10.1080/14780887.2014.915367

[cit0031] Merleau-Ponty, M. (2013). *Phenomenology of perception*. Routledge.

[cit0032] Mortazavi, S. S., Shahbazi, N., Taban, M., Alimohammadi, A., & Shati, M. (2021). Mourning during corona: A phenomenological study of grief experience among close relatives during COVID-19 pandemics. *OMEGA - Journal of Death and Dying*, 1–22. 10.1177/00302228211032736 34282960

[cit0033] Neimeyer, R. A., Klass, D., & Dennis, M. R. (2014). A social constructionist account of grief: Loss and the narration of meaning. *Death Studies*, 38(8), 485–498. 10.1080/07481187.2014.913454 24738824

[cit0034] Neimeyer, R. A., & Lee, S. A. (2022). Circumstances of the death and associated risk factors for severity and impairment of COVID-19 grief. *Death Studies*, 46(1), 34–42. 10.1080/07481187.2021.1896459 34019471

[cit0035] Neimeyer, R. A., Milman, E., & Lee, S. A. (2021). Apocalypse now: COVID-19 and the crisis of meaning. In *Death, grief and loss in the context of COVID-19*. Routledge. 10.4324/9781003125990-3-5

[cit0036] Otani, H., Yoshida, S., Morita, T., Aoyama, M., Kizawa, Y., Shima, Y., Tsuneto, S., & Miyashita, M. (2017). Meaningful communication before death, but not present at the time of death itself, is associated with better outcomes on measures of depression and complicated grief among bereaved family members of cancer patients. *Journal of Pain Management*, 54(3), 273–279. 10.1016/j.jpainsymman.2017.07.010 28711756

[cit0037] Parisi, R., Lagomarsino, F., Rania, N., & Coppola, I. (2021). Women face to fear and safety devices during the COVID-19 pandemic in Italy: Impact of physical distancing on individual responsibility, intimate, and social relationship. *Frontiers in Public Health*, 9, 1–12. 10.3389/fpubh.2021.622155 PMC7994331 33777882

[cit0038] Pattison, N. (2020). End-of-life decisions and care in the midst of a global coronavirus (COVID-19) pandemic. *Intensive & Critical Care Nursing*, 58, 1–3. 10.1016/j.iccn.2020.102862 PMC7132475 32280052

[cit0039] Paul, D., & Vasudevan, M. H. (2021). Exploring mortality salience and pandemic impact in the context of COVID-19. *OMEGA - Journal of Death and Dying*, 1–19. 10.1177/00302228211056221 34866468

[cit0040] Perin, M., & De Panfilis, L. (2021). Among equity and dignity: An argument-based review of European ethical guidelines under COVID-19. *BMC Medical Ethics*, 22(36), 1–29. 10.1186/s12910-021-00603-9 33789633 PMC8011067

[cit0041] Perron, C., Martisella Gonzalez, M., & Bouthillier, M. -È. (2022). Enjeux éthiques de la présence et de l’absence des proches auprès des personnes en fin de vie en temps de pandémie. *Frontières*, 33(1), 1–15. 10.7202/1089343ar

[cit0042] Ponterotto, J. G. (2005). Qualitative research in counseling psychology: A primer on research paradigms and philosophy of science. *Journal of Counseling Psychology*, 52(2), 126–136. 10.1037/0022-0167.52.2.126

[cit0043] Ricoeur, P. (1995). *Oneself as another*. University of Chicago Press.

[cit0044] Ricoeur, P. (2013). La Souffrance n’est pas la douleur. In C. Marin & N. Zaccaï-Reyners (Eds.), *Souffrance et douleur*. *Autour de Paul Ricoeur*. Presses Universitaires de France.

[cit0045] Robert, R., Kentish-Barnes, N., Boyer, A., Laurent, A., Azoulay, E., & Reignier, J. (2020). Ethical dilemmas due to the Covid-19 pandemic. *Annals of Intensive Care*, 10(84), 1–9. 10.1186/s13613-020-00702-7 32556826 PMC7298921

[cit0046] Smith, J. A. (2007). Hermeneutics, human sciences and health: Linking theory and practice. *International Journal of Qualitative Studies on Health and Well-Being*, 2(1), 3–11. 10.1080/17482620601016120

[cit0047] Smith, J. A., Flowers, P., & Larkin, M. (2009). *Interpretative phenomenological analysis. Theory, Method and Research*. Sage.

[cit0048] Stroebe, M., & Schut, H. (2021). Bereavement in times of COVID-19: A review and theoretical framework. *OMEGA - Journal of Death and Dying*, 82(3), 500–522. 10.1177/0030222820966928 33086903 PMC7756059

[cit0049] Totman, J., Pistrang, N., Smith, S., Hennessey, S., & Martin, J. (2015). “You only have one chance to get it right”: A qualitative study of relatives’ experiences of caring at home for a family member with terminal cancer. *Palliative Medicine*, 29(6), 496–507. 10.1177/0269216314566840 25634637

[cit0050] Tracy, S. J. (2010). Qualitative quality: Eight “big-tent” criteria for excellent qualitative research. *Qualitative Inquiry*, 16(10), 837–851. 10.1177/1077800410383121

[cit0051] Tuffour, I. (2017). A critical overview of Interpretative phenomenological analysis: A contemporary qualitative approach. *Journal of Healthcare Communication*, 2(4), 52–56. 10.4172/2472-1654.100093

[cit0052] Ullrich, A., Thoecahri, M., Bergelt, C., Marx, G., Woellert, K., Bokemeyer, C., & Oechsle, K. (2020). Ethical challenges in family caregivers of patients with advanced cancer - a qualitative study. *BMC Palliative Care*, 19(70), 1–13. 10.1186/s12904-020-00573-6 32423444 PMC7236546

[cit0053] Ummel, D., & Vachon, M. (2022). Perdre un être cher en contexte de COVID-19: le deuil pandémique comme expérience de deuil désaffranchi? *Frontières*, 33(1), 1–18. 10.7202/1089342ar

[cit0054] Ummel, D., Vachon, M., & Guité-Verret, A. (2022). Acknowledging bereavement, strengthening communities: Introducing an online compassionate community initiative for the recognition of pandemic grief. *American Journal of Community Psychology*, 69(3–4), 369–379. 10.1002/ajcp.12576 34935144

[cit0055] Vachon, M. (2014). Vers de nouveaux repères de dignité: Phénoménologie et rituel d’accompagnement en contexte de maladie dégénérative. *Jusqu’à la Mort Accompagner la Vie*, 117(2), 57–65. 10.3917/jalmalv.117.0057

[cit0056] Vachon, M. (2020a). Caring ethically of life and death: An existential perspective on palliative care and midwifery. *Journal of Nursing and Midwifery*, 1(1), 1–5.

[cit0057] Vachon, M. (2020b). “It made me more human”: The existential journeys of family caregivers from prognosis notification until after the death of a loved one. *Journal of Palliative Medicine*, 23(12), 1613–1618. 10.1089/jpm.2019.0689 32343649

[cit0058] Vachon, M., Ummel, D., Bourget-Godbout, A., Guité-Verret, A., & Laperle, P. (2020). Le Projet J’accompagne. Panser et repenser la fin de vie et le deuil à l’heure de la pandémie de COVID-19. *Les Cahiers Francophones de Soins Palliatifs*, 20(1), 1–11.

[cit0059] van Wijngaarden, E., Hanneke van der, M., & Dahlberg, K. (2017). Researching health care as a meaningful practice: Toward a nondualistic view on evidence for qualitative research. *Qualitative Health Research*, 27(11), 1738–1747. 10.1177/1049732317711133 28799478

[cit0060] Wang, S. S., Teo, W. Z., Yee, C. W., & Chai, Y. W. (2020). Pursuing a good death in the time of COVID-19. *Journal of Palliative Medicine*, 23(6), 754–755. 10.1089/jpm.2020.0198 32311289

[cit0061] Washington, K. T., Pike, K. C., Demiris, G., Parker Oliver, D., Albright, D. L., & Lewis, A. M. (2015). Gender differences in caregiving at end of life: Implications for hospice teams. *Journal of Palliative Medicine*, 18(12), 1048–1053. 10.1089/jpm.2015.0214 26484426 PMC4677542

[cit0062] Weigel, C. (2019). Caregiving and moral distress for family caregivers during early-stage Alzheimer’s disease. *International Journal of Feminist Approaches of Bioethics*, 12(2), 74–91. 10.3138/ijfab.12.2.05

[cit0063] Wiener, L., Rosenberg, A. R., Pennarola, B., Fry, A., & Weaver, M. (2021). Navigating the terrain of moral distress: Experiences of pediatric end-of-life care and bereavement during COVID-19. *Palliative & Supportive Care*, 19(2), 129–134. 10.1017/S1478951521000225 33648612 PMC7985909

[cit0064] Willig, C. (2012). *Qualitative interpretation and analysis in psychology*. Open University/McGraw Hill.

[cit0065] Wilson, D. M., MacLeod, R., & Houttekier, D. (2016). Examining linkages between bereavement grief intensity and perceived death quality: Qualitative findings. *OMEGA - Journal of Death and Dying*, 74(2), 260–274. 10.1177/0030222815598442

[cit0066] Yalom, I. D. (1980). *Existential psychotherapy*. Basic Books.

[cit0067] Yalom, I. D. (2008). *Staring at the sun. Overcoming the terror of death*. Jossey-Bass.

